# Intercultural pathways to indigenous mental health: the integration of ayahuasca in community care in the Yaqui intercultural medicine clinic in Sonora, Mexico

**DOI:** 10.3389/fpubh.2026.1918846

**Published:** 2026-10-09

**Authors:** Cecile Giovannetti, Anja Katharina Loizaga-Velder, Brian Rush, Rachel Gloria Ochoa Jaime, Seguaa Maria Garcia Valenzuela, Olivia Marcus, Gabby Agin-Liebes, Wouter Vanderplasschen

**Affiliations:** 1 Ghent University, Ghent, Belgium; 2 Nierika Institute for Intercultural Medicine, Chalmita, Mexico; 3 Centre for Addiction and Mental Health, Toronto, ON, Canada; 4 Yaqui Intercultural Medicine Clinic, Torim, Mexico; 5 Universidad del Pueblo Yaqui, Vicam, Mexico; 6 New York University, New York, NY, United States; 7 University of California, San Francisco, San Francisco, CA, United States

**Keywords:** ayahuasca, community clinic, indigenous ethics, indigenous health, intercultural mental health, Mexico, substance use disorders, Yaqui

## Abstract

**Background:**

Indigenous peoples experience substantial mental health and substance use disparities while remaining underserved by conventional healthcare systems. Despite growing evidence for psychedelic-assisted therapies, Indigenous knowledge systems and Indigenous-led models of psychedelic care remain underrepresented. This paper describes the establishment and implementation of the Yaqui Intercultural Medicine Clinic in Sonora, Mexico, an Indigenous-led, community-based program integrating ayahuasca within an intercultural therapeutic framework.

**Methods:**

We present a community case study documenting the development, therapeutic model, implementation processes, and lessons learned from the clinic. The program was developed collaboratively by Yaqui traditional authorities, local healthcare workers, Indigenous medicine practitioners, psychotherapists, and external partners. Care combined individual and group psychotherapy, ceremonial ayahuasca, sweat lodge rituals, family and community engagement, peer support, and culturally grounded activities including Yaqui language, music, dance, and land-based practices. A naturalistic mixed-methods research protocol was implemented alongside clinical care to assess safety and therapeutic outcomes. The clinic provided care to 130 patients; psychometric outcomes from 37 patients have been reported previously.

**Results:**

The implementation experience demonstrates the feasibility of culturally adapted psychedelic-assisted care within Indigenous territory when grounded in community ownership and collaboration between Indigenous and biomedical knowledge systems. Key facilitators included endorsement by traditional authorities, locally embedded staff, family and community involvement, continuous intercultural training, and intertribal knowledge exchange. The program developed multiple pathways into care and extended its focus beyond symptom reduction and abstinence toward psychosocial functioning, cultural identity, community participation, and collective wellbeing. Implementation was nevertheless constrained by geographic isolation, limited resources, workforce limitations, and legal uncertainty surrounding ayahuasca.

**Conclusion:**

The Yaqui Intercultural Medicine Clinic provides a rare example of Indigenous-led, community-based psychedelic care embedded within a broader intercultural mental health approach. To address mental health in Indigenous communities, models of care including use of psychedelic medicines requires revitalisation of Indigenous knowledge systems, authority over healthcare practices, and the right of communities to define their own pathways to healing. The case study therefore illustrates an alternative to therapeutic innovation largely situated within Western biomedical institutions, and calls for greater attention to Indigenous sovereignty, reciprocity, and historical and contemporary inequities.

## Introduction

There has been substantial progress over the past decade in generating clinical evidence and standardized protocols for the safe administration of psychedelic compounds within Western biomedical contexts, with applications for treatment-resistant depression, post-traumatic stress disorder, and substance use disorders (1, 2). Yet this rapid clinical expansion has unfolded alongside comparatively limited attention to the Indigenous knowledge systems from which many psychedelic medicines originate (3).

Numerous psychoactive compounds now investigated in clinical trials derive from plants and fungi long used within Indigenous healing traditions, creating ethical and public health responsibilities toward Indigenous populations, which are only rarely taken into account (4–6). Researchers, clinicians, and policymakers must address how benefits accruing from psychedelic use within Western contexts may contribute to addressing health disparities in Indigenous populations and may even exacerbate them. Further, there is also a need to bring visibility to Indigenous-led public health interventions making use of traditional medicine systems as they may provide valuable insights into Indigenous approaches to healing and inform the development of more culturally responsive therapeutic protocols for underserved populations (7).

Globally, Indigenous peoples experience a disproportionately high burden of mental distress, including elevated rates of depression, anxiety, trauma-related conditions, and substance and alcohol use disorders (8). At the same time, wide gaps persist for mental health treatment, including treatment for problematic substance use, reaching up to 80% in the few settings where reliable data exist (9). Barriers to care for Indigenous people include geographic isolation, linguistic exclusion, stigma, financial constraints, and health systems that often fail to integrate culturally appropriate elements such as Indigenous identity, community ties, and traditional practices as pathways to healing (10, 11). If and when mental health services are available, they tend to specialize in acute care for serious conditions such as substance use disorders, prioritizing individual-level symptom reduction while overlooking community networks, spiritual dimensions of care, and the social determinants that shape well-being.

The United Nations Declaration on the Rights of Indigenous Peoples (UNDRIP) affirms the right of Indigenous peoples to maintain traditional medicines and health practices (Article 24) and upholds their right to self-determination in matters affecting their well-being. In the context of the rapid expansion and commercialization of traditional medicine practices using psychoactive plants and fungi such as ayahuasca, peyote, and psilocybin mushrooms, Indigenous communities have advocated for greater access, visibility, and inclusion of Indigenous peoples using Indigenous medicines to address Indigenous mental health (5). Intertribal exchange such as the use of ayahuasca in a different indigenous context from the one it originates may also provide an important pathway for cultural revitalisation and healing, as highlighted by research among native American communities, where it provided some pathways for resource sharing, knowledge exchange, cultural self-confidence, capacity building and social mobilization (12, 13).

A multilevel gap continues to expand along with the development of psychedelic therapies that involves an equity gap, a reciprocity gap and an implementation gap. Indigenous populations who have contributed to the development of contemporary psychedelic therapies often face the greatest barriers to accessing mental healthcare (equity gap). The biomedical psychedelic and wellness industries in the West benefit private companies with little redistribution to Indigenous groups or acknowledgement of their profligate use of intangible cultural heritage and intellectual property (reciprocity gap). Last, the underrepresentation of Indigenous peoples in psychedelic research has resulted in persistent gaps in knowledge and advocacy regarding their needs and in the development of culturally attuned treatment models for indigenous communities (implementation gap) (14, 15). To address these gaps, Indigenous people worldwide therefore need to have priority access to the benefits offered by biomedical developments in psychedelic therapies. Increasing efforts in this direction are being made globally through the development of various psychedelic therapy models in the Global South (16, 17). Examples include an intercultural mental healthcare program designed in Canada integrating ketamine-assisted therapy (18), an Indigenous-led mental health clinic in New Zealand addressing problematic methamphetamine use among Māori people through a culturally adapted program including therapeutic use of psilocybin (19), and advocacy efforts for Aboriginal Australian people to access psychedelic therapy on their own cultural terms (20).

Psychedelic-assisted treatment initiatives designed for and/or that are accessible by Indigenous people for the treatment of mental health and substance use remain very limited. Even more limited are initiatives that exist within indigenous territories. Such initiatives must focus on how to tailor the application of psychedelic therapies to the specific cultural contexts, historical backgrounds, and clinical and psychosocial needs of Indigenous populations. Although resource-intensive, such initiatives often involve significant returns on investment on mental health outcomes for a large population compared to high-cost randomized controlled trials; Moreover the sustainable design and implementation of community mental health programs in Indigenous contexts offer various ways to inform other local programs, as well as a more equitable expansion of psychedelic therapies more broadly (21).

This paper therefore aims to present the work of the Yaqui Intercultural Medicine Clinic in Sonora, Mexico, a community-based intervention integrating ayahuasca into a culturally adapted therapeutic approach to address substance use and mental health challenges among Indigenous Yaqui patients. The design and implementation of this intervention are described with the aim of providing insights that may facilitate the development of similar programs in other Indigenous communities and contribute to addressing some of the public health needs and gaps identified above.

Previous publications from this research group have documented the clinical trajectories of selected patients (22) and psychometric outcomes among patients attending the clinic (23), reporting encouraging improvements in several mental health and substance use related outcomes. Building on these findings, the present paper focuses on the processes underlying the establishment and implementation of the clinic, thereby providing a complementary account of the intervention beyond individual outcomes.

The authors present the establishment of the clinic as a community case study, documenting the programmatic steps that led to its formation and reflecting on the application of a culturally adapted therapeutic protocol, as well as its broader implications for community strengthening and cultural revitalisation. Lessons learned from the implementation of this intervention may inform more equitable approaches to the development of psychedelic-assisted treatment programs and contribute to reparative and empowering models of care for historically marginalized communities.

## Context and setting

The Yaqui Intercultural Medicine Clinic was established in 2021 in the village of Tórim, within Yaqui territory in the state of Sonora, Mexico. The Yaqui people of Sonora number approximately 35,000 individuals and represent one of the largest Indigenous groups in northern Mexico. Their history has been characterized by sustained resistance to colonization, territorial dispossession, and resource extraction, including conflicts over land and water rights. These historical processes continue to shape contemporary social and health conditions (24).

Considering the high burden of substance-related harms experienced by many Indigenous communities worldwide (25, 26), it is important to consider how the enduring effects of violent colonization, including historical trauma, dispossession, and disruption of community structures, may have created conditions of vulnerability to the introduction of externally sourced substances such as alcohol and methamphetamine, which then compounds pre-existing cycles of intergenerational trauma and social disadvantage (27). The Yaqui territory in Sonora has experienced escalating psychosocial challenges in recent decades, including the expansion of organized criminal activity and rising methamphetamine and alcohol use, particularly among male youth. Although epidemiological data specific to the Yaqui community are limited, community leaders and health workers report increasing levels of substance use and cartel-related violence leading to family disruption, and systemic trauma. Kidnappings of young people involved in cartel networks are a regular occurrence, causing anxiety, depression, grief, and trauma among families.

In Yaqui territory, as in many Indigenous territories around the world, the spread of alcohol and other substances does not only damages individual lives through addiction, incarceration, violence, or premature mortality but may also weaken the intergenerational transmission of language, ceremonial responsibilities, and cultural heritage (28).

In addition to local cartel-related violence, Yaqui people face broader structural inequalities such as limited economic and educational opportunities and barriers to accessing health services. Professional mental healthcare services available locally are insufficient in the area. Most patients with SUD are “treated” in substance use rehabilitation centers, so called “anexos” operating under outdated, low quality, culturally unadapted and at times even coercive models with violent elements (29) often being overcrowded and catering almost exclusively to male service users. Specialist tertiary psychiatric care is available in the cities, while in rural areas the sporadic mental health care available is not adapted to the needs of the Yaqui community who face significant geographical, economic, linguistic and cultural barriers (30, 31).

## Key programmatic elements

The following section relays the conceptual basis, conditions of inception, therapeutic protocol, and capacity strengthening elements of the clinic and program, as summarized in Table 1.

**Table 1:** Summary table of key program elements.

Key programmatic elements	Key elements of the therapeutic process	Associated outcomes
Initiative requested by Yaqui traditional authorities; Indigenous governance and local leadership; external partners support rather than direct the intervention	Community ownership and trust as the foundation of care	Legitimacy and trust; community acceptance; stronger engagement and retention
Intercultural medicine framework; multidisciplinary team; collaboration between Yaqui staff, traditional authorities and Nierika clinicians and researchers	Integrating Indigenous and biomedical models in clinical practice and research	Development of a safe, culturally adapted and clinically informed model of care Dissemination of programme results for legal advocacy and indigenous health awareness
Care delivered by locally trusted Yaqui staff; outreach through community health workers and word of mouth	Establishing trust and therapeutic alliance	Improved engagement and continuity of care; greater acceptability of mental health treatment
Outpatient community-based model within indigenous territory; informal social spaces; shared meals and activities; peer relationships and family involvement	Creating a safe relational space	Patient retention; increased social participation; stronger relationships with family and peers
Family members actively involved in therapy; community health workers conducting family outreach, systemic focus	Working with family and community systems	Improved family relationships; greater community engagement; broader reach of therapeutic work and wellbeing outcomes
Combination of individual therapy, group sessions, sweat lodges, ceremonial activities, and informal peer support Integration of Yaqui cultural practices, language, music, dance, crafts and ceremonial practices Land based component to ceremonies	Creating varied culturally adapted therapeutic pathways	Development of multiple pathways into the continuum of care; Strengthening of indigenous identity and cultural revitalisation Collective sense of healing; strengthened social cohesion and community resilience
Group ayahuasca ceremonies supported by preparation, psychotherapy and integration; Sweat lodges ritual and prayer	Processing grief and trauma through ceremony	Improvement in mental health; Adoption of indigenous cultural practices fostering a sense of cultural revitalisation
Attention to psychosocial functioning and personal resources, defining recovery beyond abstinence	Transdiagnostic and strength-based approach to mental health	Functional improvements among patients mental health including
Participation in Indigenous-led gatherings, Sundance and Indigenous Ayahuasca Conference; opportunities for former patients and staff to take on peer and ceremonial roles	Strengthening capacity and developing spiritual leadership	Development of peer mentors and spiritual leadership capacities; strengthened human resource sustainability

### Conceptual basis

The conceptual basis of the Yaqui Intercultural Medicine Clinic was developed through collaborative work with the Yaqui community and is congruent with theoretical foundations established by Latin American researchers in anthropology, public health, and community health service delivery, which have been developed under the framework of intercultural medicine (32).

Intercultural medicine can be defined as an approach to health and medicine that recognizes the plurality and equal legitimacy of knowledge systems, including biomedical approaches and Indigenous models, and seeks to integrate them through interactions based on mutual respect, willingness to learn from one another, and openness to dialogue in service of populations (33). Through the intercultural lens, healthcare services must be culturally acceptable to the communities they serve, including language accessibility, recognition of local beliefs, incorporation of community values, accommodation of traditional practices, and reduction of discrimination within healthcare systems. The World Health Organization (WHO) defines cultural acceptability as a core component of Indigenous peoples’ right to health (34). In the context of the Yaqui clinic, continuous dialogue between the clinic’s Indigenous staff and the research and clinical supervision team, as well as regular consultation with traditional authorities, was integrated into program design and evaluation, fostering a collaborative approach.

An intercultural medicine approach extends beyond focus on physical or mental symptoms to include the health of the community, land, and territory, as well as cultural, social, and spiritual dimensions of health (35). This expanded perspective prompted the clinic to go beyond the delivery of psychotherapeutic services and include cultural revitalization, community engagement, and strengthening of cultural leadership, with the goal of developing a holistic model beyond clinical outcomes and toward a broader community public health intervention.

Much of the theoretical and empirical basis for integrating ayahuasca into mental health treatment, particularly for substance use disorders, was laid by the pioneering work of the Takiwasi Center. Takiwasi is a residential clinic and therapeutic community for substance use rehabilitation in the Peruvian Amazon, founded in 1992, which has treated over 1,000 patients through a model integrating ayahuasca, traditional Amazonian medicine, and psychotherapy in a 3–9-month therapeutic program (36, 37), with reported reductions in addiction severity and co-occurring conditions (38).

While the Yaqui Intercultural Medicine Clinic is unique in its focus on serving a specific Indigenous community, the ritual use of ayahuasca combined with structured psychotherapeutic support for the treatment substance use disorders was developed, refined and tested for decades at the Takiwasi Center in Peru. Recent observational studies among Takiwasi patients suggest reductions in addiction severity and co-occurring conditions in both long and short term stays (38). The Yaqui clinic model drew from the evidence base and clinical expertise of the Takiwasi center and the Nierika Institute for intercultural medicine in integrating the therapeutic use of ayahuasca and other practices from indigenous medicine traditions in substance use and mental health treatment, while adapting to the local Yaqui context through an initial participatory needs assessment.

### Inception of the clinic

In response to the on-going mental health and substance use crises in their community, a group of young health professionals and traditional practitioners with support of the traditional Yaqui authorities contacted the Interdisciplinary Association for the Preservation of Indigenous Traditions Involving Sacred Plants in the Americas (Nierika A. C.), a non-profit organization whose mission is to support the research and biocultural preservation of Indigenous traditions, rituals, and ceremonial practices involving sacred plants. In addition, the organization contributes to the development of regulatory frameworks for the use of these plants in intercultural therapeutic contexts in Mexico and operates since 2010 an intercultural therapeutic program for addictions and mental health challenges.

Following a request from a group of young Yaqui healthcare staff and with the support of the traditional authorities of the Yaqui people, Indigenous medicine holders from different Amazonian Indigenous groups (including Huni Kuin & Noke Koi) along with representatives from the Nierika association traveled to Yaqui territory monthly to bimonthly for 2 years to offer ayahuasca ceremonies for members of the Yaqui tribe with mental health and substance use challenges including methamphetamine and alcohol use, trauma depression, anxiety and complicated grief. The traditional authorities considered, based on their own experience with ayahuasca, that this form of traditional medicine, while it originated from another Indigenous group, was an Indigenous medicine that could be a “spiritual vitamin” to address the ailments of their community’s spirit. As methamphetamine use and related social harms were widely understood as challenges introduced from outside Yaqui culture and territory, Yaqui elders viewed the adoption of an Indigenous medicine from another tradition as a culturally coherent response to these emerging forms of distress. For this reason they supported r the establishment of the Yaqui intercultural medicine clinic.

Participatory methods were used by a team of clinicians and researchers alongside the Yaqui clinic team to develop a medically, psychologically and interculturally informed protocol guiding the implementation of group-based ayahuasca ceremonies within preparatory and integrative psychotherapeutic processes.

This participatory process involved Indigenous leadership in defining the need for the intervention, local staff and traditional authorities in developing and adapting the protocol, and the integration of Indigenous, biomedical and psychological knowledge through ongoing clinical review. Patient and family input, the involvement of community health workers, and continuous supervision and collective reflection further supported the iterative adaptation of the program to community needs and context.

As a result, the Yaqui Intercultural Medicine Clinic was conceived as an outpatient, community-based clinic providing culturally adapted and integrated care to Yaqui community members with varied demographic and clinical profiles. Drawing on the therapeutic model of Takiwasi in the integration of group ritual use of ayahuasca within a culturally tailored and structured psychotherapeutic intervention, the Yaqui clinic model differed by offering outpatient community service to improve access for patients by allowing them to continue their work and family commitments while attending treatment locally. Further, the Yaqui program was designed to include patient’s family members as active participants in the recovery process. This strengthened treatment engagement and continuity while acknowledging that mental health difficulties and recovery are embedded within family and community dynamics. By extending support beyond the individual patient, the clinic amplified its impact across wider family and community networks.

The original staff numbering six included a Yaqui-mestizo psychotherapist, a mestizo psychotherapist, a mestizo medical doctor, a Yaqui nurse, a Yaqui administrative and research staff, and one Yaqui community health worker. The team was able to work both in Spanish and in Yaqui language and maintaining a strong existing relationship of trust with the local community. Funding for the clinic’s staff and activities was secured though the support of several philanthropic foundations supporting the preservation and ethical integration of traditional medicines.

Staff from the Nierika association—composed of clinical psychotherapists, anthropologists, and Indigenous rights activists— activists collaborated with the Yaqui team over a period of 12 years to develop a culturally attuned treatment program which served as a complementary therapeutic offer for patients apt for these interventions and included the intercultural application of ayahuasca in traditional ceremonial ways. Nierika AC also provided in-depth training in providing psychotherapeutic support around the intercultural integration of ayahuasca in an Indigenous context through a one-month residential training program, followed by ongoing monthly clinical and administrative supervision.

A timeline of key events in the establishment of the clinic is provided in Appendix 2.

### Therapeutic protocol

The therapeutic protocol of the Yaqui Intercultural Medicine Clinic was designed to provide safe, effective, and culturally attuned mental health and substance use treatment and support to Yaqui patients.

Patients entered treatment through outreach activities conducted by community health workers and through word of mouth. An initial assessment was performed by a psychotherapist to evaluate the patient’s mental health needs and capacity to engage in treatment, including geographic, economic, and social constraints. A medical evaluation was also conducted by the clinic’s physician. Detailed information regarding the treatment program and optional ayahuasca interventions was provided both orally and in written form. Upon entry, a clinical file was created, including the patient’s medical history and psychotherapy notes.

The initial treatment stage, lasting approximately 1 to 3 months, focused primarily on establishing a therapeutic alliance and building capacity for treatment continuity through a combination of individual psychotherapy and group activities in the clinic (sports, shared meals, etc.) During the whole treatment spam monthly sweat lodge rituals were held to provide ritualized spaces for prayer and community healing, and local traditions such as Yaqui dances, music, and crafts were also practiced on the clinic’s grounds.

The second phase of treatment focused on identifying causes of distress, developing strategies for self-regulation, and addressing environmental stressors and risk situations in family or workplace contexts. Patients who wished to attend the monthly ayahuasca ceremonies and passed through screening for counter indications could also attend this complementary therapeutic offer.

Individual psychotherapy was complemented by group preparation and integration sessions before and after the ayahuasca ceremonies. After 6 months to 1 year of treatment, or as jointly decided by the psychotherapist and the patient, patients entered the final treatment stage, focused on consolidating a healthy self-image and building individual and community resources to maintain recovery. After a final assessment, patients were discharged with the possibility of maintaining occasional psychotherapy or participating in community activities, sweat lodges, ayahuasca ceremonies, and were encouraged to continue visiting the clinic to engage with peers in recovery. Some of the recovered patients trained to become peer counselors and were integrated as part of the therapeutic team. Due to their economic situation, most patients could not afford being away from work while receiving addiction care. This prompted the clinic’s staff to develop the opportunity for some patients to get paid work on the clinic’s ground temporarily to provide a substance-free work environment.

While the protocol initially centered on formal psychotherapeutic interventions, on-going reflection with staff and patients highlighted the importance of multiple pathways of care. For many, discussing personal matters remained culturally sensitive, even within a strong therapeutic alliance. Some patients benefited more from the informal relationship with peers in recovery and partaking in practical activities such as building or cleaning the clinic grounds. Others found greater comfort in informal social spaces such as the family home from which the clinic operated and where meals were shared. Some found the sweat lodge to be a space for prayer, healing, and mourning, enabling reflection on experiences that were difficult to express in formal therapy. Over time, the role of clinic staff increasingly shifted toward facilitating a safe and supportive community environment in which patients could develop a shared identity as fellow Yaqui tribe members.

A central theme emerging from patients’ lived experiences and underlying the depression, anxiety, addictions and PTSD symptoms was profound grief related to multiple losses: Yaqui ancestors who died defending their lands, family members lost to methamphetamine use, kidnappings linked to cartel violence, the COVID-19 pandemic, and the loss of land, wildlife, and the Yaqui River. Ayahuasca experiences often provided a ceremonial container in which patients reported symbolic encounters with deceased relatives or ancestors, facilitating processes of resolution and acceptance. Another common theme was a strong sense of reconnection with ancestral land and cultural identity, which provided comfort and strength. The collective use of Indigenous medicine appeared to reinforce cultural identity, which emerged as an important therapeutic mechanism in recovery. For illustrative case studies, see Loizaga-Velder et al. (22).

### Research protocol

In addition to the therapeutic program, a mixed-methods naturalistic longitudinal research protocol was developed with an international team to assess the safety and effectiveness of the intervention. Given the specific population focus of the clinic, the research component also aimed to generate evidence to support reflection on clinical trajectories and best practices, as well as to contribute to the evidence base on traditional medicine in Indigenous mental health and substance use treatment and support.

Patients who provided informed consent were assessed at treatment entry, before and after each ayahuasca session, and at discharge. The protocol included psychometric instruments validated for Spanish-speaking and Indigenous populations and previously used in ayahuasca-assisted treatment studies (39), including Beck’s Depression and Anxiety Inventories and the Addiction Severity Index. A Complicated Grief scale was also included, along with a peer evaluation form allowing patients’ relatives or peers to report perceived changes in mental health and social functioning. This tool also supported community health workers in conducting family outreach and psychoeducation, strengthening the integrative nature of the program. Qualitative interviews, based on guidelines co-developed with clinic staff, complemented quantitative measures.

This pilot project provided care to 130 patients, with encouraging therapeutic outcomes observed across the program. Psychometric data from 37 patients who attended at least two ayahuasca ceremonies were collected and reported in a previous publication (23), providing preliminary evidence of positive outcomes within the clinic’s broader therapeutic model. Four adverse events were monitored and managed and are reported in Appendix 1.

### Capacity strengthening

An important evolution in the clinic’s work was the gradual expansion from exclusively delivering a clinical service toward a broader model of community strengthening. Recognizing that substance use, depression, anxiety, grief, and trauma are shaped by social and historical determinants, the clinic increasingly sought to support not only individual recovery but also the development of sustainable community resources for resilience while strengthening Yaqui cultural values.

Ayahuasca ceremonies acted as a central element in helping patients and staff connect to hope and spiritual strength, which, when shared during group integration sessions, reinforced a collective sense of healing and supported the reactivation of self-regulation at both individual and community levels.

Intertribal collaboration constituted an important component of the clinic’s development. Visits from Indigenous groups such as the Huni Kuin and Noke Koi, who conducted ceremonies with clinic participants, fostered a sense of belonging within a broader Indigenous network of traditional medicine practitioners. Participation in successive Indigenous Ayahuasca Conferences further supported capacity strengthening through peer learning, knowledge exchange, and dialogue with Indigenous leaders engaged in the preservation, governance, and intercultural application of traditional medicines.

Several members of the clinic team and former patients also participated in the Sundance, a ceremonial cycle of prayer, dance, and community commitment led by Lakota elders in Mexico. Some completed a four-year cycle of participation, deepening their engagement with Indigenous spiritual traditions and strengthening their capacity to support ceremonial activities, reinforcing their roles as credible peer mentors and role models for individuals in recovery from an indigenous cultural standpoint.

## Discussion, lessons learned, and considerations on reproducibility

Overall, the care model of the Yaqui intercultural medicine clinic, integrating ayahuasca ceremonies and community engagement, proved to be a culturally adapted and clinically relevant approach to local needs. Psychometric evaluation and on-going community consultation showed that the therapeutic program provided safe and well-accepted care to patients. Preliminary outcome data indicated substantial improvements in mental health, substance use and overall functioning among many participants. Beyond symptom improvement, the clinic demonstrated the feasibility of delivering a culturally grounded mental health program within Indigenous territory through collaboration between community leadership, traditional medicine practitioners, and healthcare professionals. It appears that the potential benefits of such an approach may also extend beyond individual behavioral health outcomes. By strengthening engagement with traditional knowledge, family and community networks, and community relationships, the clinic’s work may contribute to cultural revitalisation, social cohesion, and community resilience. These broader processes may in turn reinforce individual recovery by strengthening the social and cultural resources available to patients beyond the clinical setting, potentially supporting the durability of therapeutic gains over time.

### Facilitators and barriers to implementation

Several factors facilitated the successful implementation of the clinic. First, the endorsement of traditional Yaqui authorities conferred legitimacy on the program and fostered trust among community members. Rather than introducing an externally designed intervention, the clinic emerged through dialogue with community leaders and was implemented by Yaqui psychotherapists, health workers, and administrative staff who already held established relationships within the community. This local ownership proved essential for patient and family engagement and retention and helped overcome initial resistance, for example from local religious groups.

Continuous training and capacity building also constituted an important enabling factor. Ongoing collaboration between the Yaqui team and the Nierika association provided technical expertise in program development, safety, therapeutic practice, and research implementation. At the same time, participation in Indigenous-led gatherings such as the Sundance and the Indigenous Ayahuasca Conference strengthened the clinic’s human resources through forms of learning rooted in Indigenous epistemologies. Because these occurred on a recurring basis, they also created long-term horizons for engagement, reinforcing commitment to the clinic’s mission and supporting the emergence of peer mentors with spiritual leadership skills, thereby strengthening the long-term sustainability of the initiative from a human resources development perspective.

Finally, an important facilitator of implementation was the legal exception granted by the Mexican government to allow ayahuasca use by the Yaqui, as part of the Plan for Justice for the Yaqui people, a government initiative aimed to make amends for the historical violence perpetrated against the Yaqui people. The project received approval from the National Commission on Mental Health and Addictions of the Mexican Health Department, establishing an important precedent for the supervised use of ayahuasca within an Indigenous community mental health program. This regulatory exemption allowed the intervention to be implemented within an ethical and transparent legal framework while contributing to broader discussions on the integration of traditional medicines into public health systems.

When considering possible transferability of the clinic’s model, it is worth noting that Several Latin American countries have established legal or policy frameworks recognizing traditional and Indigenous medicines and, in some cases, protecting their use by Indigenous peoples (40). These frameworks may create important opportunities for the development of intercultural mental health and substance use treatment programs; however, legal recognition does not necessarily translate into clear pathways for the integration of Indigenous medicines into formal service delivery models often requiring procedures and processes that are complex to navigate and requiring multilevel authorisation and accreditation. Furthermore, the existence of a supportive legal framework does not ensure that Indigenous communities have access to the resources, infrastructure, training, or institutional partnerships required to develop and sustain such programs.

Unfortunately, the legal status of ayahuasca in Mexico remains undefined and while the clinic was operating, several people were imprisoned for introducing ayahuasca to Mexico creating a complex legal context for continued work at the clinic while the Justice Department was also prosecuting indigenous medicine practitioners and other individuals for possession of ayahuasca. The National Council on Mental Health and Addictions requested the suspension of the intercultural clinic until the legal status of ayahuasca be clearly defined within Mexican law. However, intercultural mental health interventions continue to this day primarily through the implementation of sweatlodge ceremonies and community building activities.

In this context, findings from the clinic may support advocacy for Indigenous rights to access and practice traditional medicines. Notably, the scientific publications of observational data from the Yaqui program (22, 23) were recently submitted as supporting evidence in a legal case involving a Noke Koi traditional medicine practitioner, who was subsequently exonerated by the Mexican justice system. This illustrates the potential contribution of community-based research to policy development and the legal recognition of Indigenous health practices in Mexico and beyond.

At the same time, several barriers limit the program’s reach, reproducibility, scalability, and sustainability. The clinic operated in a resource-constrained environment characterized by poverty, geographic isolation, transportation difficulties, and the influence of organized crime. Many patients faced unstable living conditions, as well as ongoing substance use within family networks and working environments and/or ongoing exposure to violence, all of which complicated treatment engagement and continuity. The clinic’s location outside the public transport network was a barrier to access, and with a small team and only one vehicle available, outreach capacity remained limited, contributing to patient attrition during early stages of treatment.

Another limitation concerned the research activities. Given the clinic’s limited resources and the priority placed on ensuring continuity and quality of clinical care, research procedures were necessarily secondary to patients’ therapeutic needs. Consequently, staff were not always able to enroll all eligible patients in the research protocol, complete all scheduled assessments, or obtain follow-up data at every time point. This resulted in a smaller and potentially less complete research sample than intended and limited the extent to which treatment trajectories could be systematically evaluated. Given the absence of a control group, results presented in our previous publication (23) cannot establish the specific contribution or efficacy of ayahuasca within the broader intervention. Improvements in patient outcomes may also reflect the benefits of receiving care within a culturally attuned program, supported by a motivated intercultural clinical team and other components of the therapeutic intervention. Consequently, the observed improvements should be interpreted as preliminary evidence concerning the potential value of the overall care model rather than as evidence of the specific effects of ayahuasca.

When considering scalability and reproducibility, the most significant challenge lies in human resource development. Delivering an intercultural mental health and substance use treatment intervention requires a multidisciplinary team with established trust within the community and the capacity to navigate both Indigenous and biomedical knowledge systems. These combined competencies remain relatively rare, constituting a key bottleneck for scaling this model across different settings. At the same time, this challenge also highlights an important opportunity: strengthening Indigenous-led initiatives to improve access to education and ongoing professional development, alongside building collaborations with diverse partners, including other Indigenous leaders, universities, government organizations, and funding bodies.

Overall, the work of the Yaqui clinic illustrates how psychedelic-assisted interventions can move beyond Western biomedical models and specialist psychiatric settings to become embedded within community mental health systems, where their potential benefits may extend beyond symptom reduction to encompass cultural revitalisation, social cohesion, and community resilience. The underlying principles of this intervention are transferable across different Indigenous contexts, although specific practices require adaptation to local resources and needs. Mental healthcare staff considering implementing a similar intervention in another setting may consider using a different psychedelic compound or integrating culturally relevant practices for local populations. Crucially, the foundation of such interventions must be mutual trust and respect between collaborators, traditional authorities, and patients in order to maintain the ethical integrity of the work.

## Conclusion

Overall, this community case study contributes to public health practice, research, and policy by documenting a real-world, innovative model of intercultural mental health care implemented within an Indigenous population. Based on the existing literature (18, 19, 41–43), there are few documented examples of Indigenous-led mental health initiatives integrating psychedelic plant medicines within routine community-based care, making this a novel and insufficiently studied model of public health intervention. Published examples typically integrate psychedelic modalities using synthetic compounds, such as ketamine or psilocybin, within ceremonial frameworks developed through dialogue with elders and patients. However, the integration of ayahuasca into models of care involves living traditions originating within Indigenous communities, which constituted a key therapeutic element in the Yaqui clinic.

The emerging literature on Indigenous psychedelic and plant medicine initiatives consistently highlights the importance of sustained dialogue with Indigenous elders, community leaders, service providers, and patients in the development of culturally responsive models of care (5, 44, 45). Transferable lessons emerging from the implementation of the Yaqi clinic program show that community ownership, adaptation to local context and continuous capacity building have been key factors in establishing trust, fostering engagement, and ensuring cultural relevance and sustainability throughout implementation.

The experience of the Yaqui Intercultural Medicine Clinic suggests that Indigenous self-determination may be a critical enabling condition for the successful integration of traditional medicine within community mental health services. Increasing the visibility of such initiatives through Indigenous gatherings, and international conferences and academic publications may facilitate knowledge exchange between communities facing similar mental health challenges. In turn, this may support other Indigenous groups in exploring, adapting, and developing locally led models of intercultural mental healthcare that respond to their own cultural, social, and territorial realities.

In terms of public health practice, the Yaqui Intercultural Medicine Clinic demonstrates the feasibility of integrating traditional medicine, including ceremonial ayahuasca use, within a structured community mental health service delivered by a locally embedded multidisciplinary team. Beyond individual clinical outcomes, the program over its 5 years of implementation illustrates how culturally grounded mental healthcare can strengthen community outcomes, including participation, family engagement, cultural identity, and peer support networks. From a research perspective, the clinic contributes to a still limited evidence base on Indigenous-led mental health interventions incorporating traditional medicines within naturalistic community settings.

More broadly, this community case study invites reflection on the direction of the contemporary psychedelic field. While much attention has focused on the development of novel clinical treatments within specialist healthcare settings, the experience of the Yaqui community suggests that psychedelic and traditional medicine interventions may also have a role within community-based models of care that address the social, cultural, spiritual, and collective dimensions of mental health. The lessons emerging from this initiative extend beyond the therapeutic use of ayahuasca, highlighting the importance of Indigenous self-determination, cultural continuity, and community ownership in the design of mental health services. As global interest in psychedelic therapies continues to expand, supporting Indigenous-led models of care may also contribute to addressing the historical and structural conditions that continue to shape Indigenous mental health today. Initiatives such as the Yaqui Intercultural Medicine Clinic offer valuable perspectives on the broader evolution of psychedelic-assisted care, emphasizing the importance of community, culture, spirituality, and collective healing.

## Data Availability

The original contributions presented in the study are included in the article/supplementary material, further inquiries can be directed to the corresponding author.
