# Supplementary material for: Intercultural pathways to indigenous mental health: the integration of ayahuasca in community care in the Yaqui intercultural medicine clinic in Sonora, Mexico
Source: Front Public Health. 2026 Oct 9;14:1918846. doi: 10.3389/fpubh.2026.1918846 (PMC13690547; doi:10.3389/fpubh.2026.1918846)
Supplement: Supplementary file 1 [file Supplementary_file_1.pdf]

Appendix 1: Reports on adverse events and their clinical management and programmatic response

Case 1: One participant was a family member of a clinic patient and joined the ayahuasca ceremony as part of the family support network of that patient, which was part of the clinic's community care model. Being part of a family of traditional healers and a trusted community member, she had not gone through the in-depth screening procedure (lasting 2+ hours). However, having a history of complex trauma and being a highly sensitive person, she remained in a dissociated state after the ceremony ended. The symptoms she presented with shared elements of psychotic symptoms mixed with elements of energetic ailments when considered through a traditional medicine lens, and the care she received was therefore intercultural: firstly through family care, the intervention of a traditional medicine doctor, and antipsychotic medication prescribed by a psychiatrist, which she took over 10 days until the symptoms resolved.

Case 2: One patient relapsed into methamphetamine use after an ayahuasca ceremony during which he was confronted with the repressed emotions tied to the assassination of his brother, which he had witnessed. The overwhelming intensity of this experience triggered him to seek methamphetamine use as a way to cope with the grief that he was now able to be in touch with. This resulted in a consultation with the clinical team to ensure that patients confronted with intense emotions during ceremonies received additional care.

Case 3: One patient had difficulty sleeping for two nights after an ayahuasca ceremony and became agitated. She was offered herbal medicine for sleep and psychological support until she was able to sleep again.

Case 4: After an ayahuasca ceremony, one patient became more vulnerable to the harmful culture of the work environment in which they were working, where methamphetamine use was widespread, causing both a relapse and the inability to continue working there. He was offered temporary paid work on the clinic's grounds and additional psychotherapeutic support.

Appendix 2: Timeline of key events in the establishment of the clinic

2014–2015 – Various Indigenous traditional healers visited Yaqui territory and offered peyote and ayahuasca ceremonies to community members experiencing problematic methamphetamine use.

January–September 2020 – Yaqui Traditional Authorities initiated discussions with the Sonora State Department of Public Health regarding the establishment of an Intercultural Medicine Clinic. With technical support from Nierika A.C., a project proposal for a community-based mental health clinic was developed and presented first to the Sonora State Department of Public Health, and subsequently to the National Commission on Mental Health and Addiction (CONASAMA) and the Riverstyx Foundation for potential project funding.

October 2020 – As part of the *Plan de Justicia para el Pueblo Yaqui* (Plan for Justice for the Yaqui People), a national restorative justice initiative addressing historical injustices and dispossession experienced by the Yaqui people, the President of Mexico announced government support for the establishment of the Yaqui Intercultural Medicine Clinic.

March 2021 – The Mexican Federal Department of Public Health and the Riverstyx Foundation committed financial and institutional support to the establishment of the clinic.

April 2021 – Yaqui–Huni Kuin intertribal encounters and training activities for the Yaqui clinical team took place.

May–August 2021 – An international research team was assembled to develop a research protocol to evaluate outcomes associated with the clinic's programme.

2022 – The clinic began receiving patients and providing psychotherapy, sweat lodge ceremonies, and other forms of community-based care.

March 2023 – The clinic began integrating ayahuasca ceremonies into its therapeutic programme.
